# A Chemosensory Protein Detects Antifeedant in Locust (*Locusta migratoria*)

**DOI:** 10.3390/insects12010001

**Published:** 2020-12-23

**Authors:** Xingcong Jiang, Haozhi Xu, Nan Zheng, Xuewei Yin, Long Zhang

**Affiliations:** Department of Grassland Resources and Ecology, College of Grassland Science and Technology, China Agricultural University, Beijing 100193, China; xjiang@ice.mpg.de (X.J.); twt1102@163.com (H.X.); zhengnan9089@163.com (N.Z.)

**Keywords:** antifeedant, chemosensory protein, locust, feeding behavior, *Locusta migratoria*

## Abstract

**Simple Summary:**

Chemosensory proteins (CSPs) in insects are small compact polypeptides which can bind and carry hydrophobic semiochemicals. CSPs distribute in many organs of insect and have multiple functions. In chemosensory system, CSPs are thought to be responsible for detecting chemical signals from the environment. In this study, we proved that *Lmig*CSPIII, a CSP in *Locusta migratoria* is involved in detecting an antifeedant. *Lmig*CSPIII exhibits high binding affinity to α-amylcinnamaldehyde, a natural compound from non-host plant which was subsequently demonstrated to be an effective antifeedant. Knockdown of *Lmig*CSPIII gene by RNA interference showed reduced sensitivity to α-amylcinnamaldehyde but showed no changes in their physiological development or food consumption. Our findings provided new evidence that CSPs can detect antifeedant in chemosensory system of insects.

**Abstract:**

Chemosensory system is vitally important for animals to select food. Antifeedants that herbivores encounter can interfere with feeding behavior and exert physiological effects. Few studies have assessed the molecular mechanisms underlying the chemoreception of antifeedants. In this study, we demonstrated that a chemosensory protein (CSP) in *Locusta migratoria* is involved in detecting an antifeedant. This CSP, *Lmig*EST6 (GenBank Acc. No. AJ973420), we named as *Lmig*CSPIII, expressed in sensory organs where chemosensilla are widely distributed. Fluorescent binding experiments indicated that *Lmig*CSPIII exhibits high binding affinity to α-amylcinnamaldehyde (AMCAL), a natural compound from non-host plant. This compound was subsequently demonstrated to be an effective antifeedant to locusts in feeding bioassay. By injection of double-stranded RNA (dsRNA) of *Lmig*CSPIII, we generated *Lmig*CSPIII knockdown locusts. The feeding behaviour assays demonstrated that the *Lmig*CSPIII knockdown locusts had reduced sensitivity to the antifeedant but showed no changes in their physiological development or food consumption. Therefore, we inferred that this chemosensory protein is involved in antifeedant detection.

## 1. Introduction

Taste is more critical for herbivores when selecting food compared with scent [1,2,3]. Similar to the taste spectrum of mammals, insects can recognize four basic tastes: sweetness, sourness, saltiness, and bitterness [1,4]. Insect survival requires recognition of edible substances as well as compounds that may be toxic. Unpalatable plant secondary metabolites, called antifeedants, can exert deterrent effects at very low levels. Depending on the chemosensory system, insects detect feeding stimulants and feeding deterrents in plant tissues with different levels [5,6].

The chemosensory organs of insects are the hair-like sensillum, mostly on the antenna and mouthparts. Within the hair-like organs, several classes of proteins are believed to play key roles in first step of detection of chemicals, such as odorant receptors (ORs), sensory neuron membrane protein (SNMP), gustatory receptors (GRs), ionotropic receptors (IRs), odorant-binding proteins (OBPs), chemosensory proteins (CSPs), and odorant-degrading enzymes (ODEs) [4,7,8,9,10,11]. ORs, a class of seven-transmembrane domain receptors on the dendritic membrane of chemosensory neurons, are responsible for transferring chemical signals into neuronal activation [12,13,14]. OBPs are hypothesized to be carriers that bind and transport hydrophobic odorants to the ORs. ORs and OBPs are believed to detect volatiles, including those of host plants [15]. For gustation, GRs have been identified in the gustatory organs, and their functions regarding recognition of contact chemicals have been studied [16,17,18]. Additionally, CSPs, are found within the gustatory organs of insects and other invertebrates [9,19,20,21]. However, CSPs are proposed to have highly different functions, such as leg regeneration, embryo development, pheromone release and detection [22,23]. In the gustatory organs, CSPs are thought to be responsible for food selection [24].

To date, several CSPs have been identified in the migratory locust, *Locusta migratoria* [20,25,26,27,28]; they show very high sequence similarities and are expressed in chemosensory organs, such as antennae, palps, tarsi and ovipositor. Immunolocalization studies have confirmed that they exhibit specialized expression patterns in gustatory sensation-associated organs [29,30,31]. Therefore, we proposed that certain CSPs in locust might play roles in gustation. We chose *Lmig*EST6, which is an orthologous with CSPSgre-III-1 in desert locust, *Schistocerca gregaria*, named it *Lmig*CSPIII and demonstrated its involvement in detecting an antifeedant. Our results provide an insight into the molecular mechanisms underlying herbivores’ chemosensation of antifeedants.

## 2. Materials and Methods

### 2.1. Animals 

Locusts (*L. migratoria*) were crowd reared at 28–30 °C, with 60% relative humidity, and a light: dark photoperiod of 18:6 h, at College of Grassland Science and Technology, China Agricultural University, Beijing, China. The size in a cage of 100 cm × 50 cm × 50 cm with the number is 500. Fresh corn leaves were provided daily. 

### 2.2. Reverse Transcription PCR (RT-PCR) and Quantitative Real-Time Reverse Transcription PCR (qRT-PCR)

Total RNA was extracted from target tissues using Trizol reagent (Invitrogen, Carlsbad, CA, USA), following the manufacturer’s protocol. Reverse transcription was performed using the Quant cDNA Synthesis Kit (Tiangen, Beijing, China) with 1 µg of unpurified total RNA as a template in a 20 μL total volume.

RT-PCR (Promega, Madison, WI, USA) was used to assess the temporal and spatial expression profiles of *LmigCSPIII*. Primers used are shown in Table 1. The thermal cycling conditions for RT-PCR were as follows: 45 min at 45 °C and 3 min at 95 °C; followed by 30 cycles of 30 s at 95 °C, 30 s at 55 °C and 45 s at 68 °C. The reaction was completed with 10 min at 68 °C.

Both RT-PCR and qRT-PCR were used to determine RNA interference efficiency. Primers for qRT-PCR were specifically designed (Table 1). The actin gene was used as an endogenous control to correct for sample-to-sample variation. The 20 μL reaction system included 10 μL SuperReal PreMix SYBR Green (Tiangen, Beijing, China), 0.6 μL qRT-PCR Sense Primer, 0.6 μL qRT-PCR Antisense Primer, 1 μL synthesized cDNA, 2 μL ROX and 5.8 μL RNase-free H_2_O (Tiangen). The thermal cycling conditions for qRT-PCR were 15 min at 95 °C; followed by 40 cycles of 10 s at 95 °C, 20 s at 58 °C and 31 s at 72 °C. Each sample reaction was repeated three times and the results were averaged. 

### 2.3. The Expression and Purification of the Recombinant CSP

Gene-specific primers were designed for *Lmig*CSPIII subgroup (Table 1). PCR products were first cloned into the pET28a (+) plasmid vector (Novagen, Madison, WI, USA), and then sequenced to verify accurate insertion. The recombinant vector was transformed into BL21 (DE3) *Escherichia coli* cells (Novagen). The expression of the recombinant protein was induced by adding Isopropyl β-D-1-Thiogalactopyranoside (IPTG) to the culture at a final concentration of 0.4 mM, after the OD_600_ of the culture reached 0.6–0.8. Bacterial cells were harvested by centrifugation and lysed by sonication. The soluble supernatant and inclusion bodies were then analyzed by sodium dodecyl sulphate polyacrylamide gel electrophoresis (SDS-PAGE) to determine fraction that contained the protein. The majority of the recombinant protein was present in the supernatant; therefore, anion-exchange resin DE-52 (Whatman, Maidstone, UK) was used for purification, followed by gel filtration on Superose-12, as previously described [25]. Purified recombinant protein was used to prepare polyclonal antibodies for Western blotting or was used in binding experiments.

### 2.4. Western Blotting

Antiserum was obtained by injecting an adult rabbit intramuscularly with 500 µg of recombinant protein, followed by two additional injections of 300 µg after 15 and 30 days. The protein was emulsified with an equal volume of Freund’s complete adjuvant for the first injection and incomplete adjuvant for further injections. Animals were bled 10 days after the last injection and the serum was used without further purification. The specificity of antiserum of *Lmig*CSPIII are tested with all the CSPs cloned in our lab. 

After electrophoretic separation under denaturing conditions by 14% SDS-PAGE, protein extracts of locust tissues or recombinant proteins were electroblotted onto a nitrocellulose membrane. After being treated with 0.2% dried milk/0.05% Tween 20 in PBS for 1 h, the membrane was incubated with the crude antiserum against the protein at a dilution of 1:1000, and then incubated with goat anti-rabbit IgG-horseradish peroxidase conjugate (dilution 1:10,000). Immunoreactive bands were detected by treatment with 4-chloro-1-naphthol. An intermediate step using PIG (preimmune goat serum) was conducted to prevent a specific binding of the goat anti-rabbit IgG-horseradish.

### 2.5. Fluorescent Binding Assay

Compounds (Table 2) used in the binding assay were purchased from Sigma (St. Louis, MO, USA) and were of reagent grade. Ligand binding specificity was measured with a fluorescent assay on an FS-55 emission fluorescence spectrometer (Perkin-Elmer, Waltham, MA, USA), as previously described [32]. The affinity of semiochemicals was evaluated in individual competitive binding assays using 2 mM N-phenyl-1-naphthylamine (1-NPN) as the fluorescent reporter. The recombinant protein was dissolved in 50 mM Tris-Buffer (pH 7.4) and titrated with aliquots of 1 mM methanol solutions of the ligands to final concentrations of 1–20 mM. The florescent probe 1-NPN was excited at 337 nm and emission spectra were recorded between 380 nm and 440 nm. We used Graphpad Prism 5 software (Graphpad Software, San Diego, CA, USA) to calculate dissociation constants and draw the binding curves [33]. Dissociation constants of the competitors were calculated from the corresponding [IC_50_] values (concentrations of ligands that halved the initial fluorescence value of 1-NPN) using the equation: K_D_ = [IC_50_]/(1 + [1-NPN]/K_1-NPN_), where [1-NPN] indicates the free concentration of 1-NPN and K_1-NPN_ indicates the dissociation constant of the complex *Lmig*CSPIII/1-NPN.

### 2.6. Testing the Role of α-Amylcinnamaldehyde in Locust Feeding

New emerged fourth instar locust nymphs were starved 2 h before being introduced into a transparent plastic box (14 cm diameter, 8 cm high). For the non-choice experiment, the locusts were then supplied with two pieces of corn leaf (3 × 5 cm) of the same weight. One piece of the leaf was treated with an acetone solution of α-amylcinnamaldehyde (AMCAL) after the acetone evaporated completely, and the control leaf was just treated with same amount of actone. The natural concentrations of AMCAL found in non-host plants is usually less than 0.1% in weight. According to this, acetone solutions of AMCAL at concentrations of 0.5 M, 0.1 M, 0.05 M, 0.01 M, and 0.001 M were used to treat corn leaves with a final concentration of 100 μg/cm^2^, 50 μg/cm^2^, 10 μg/cm^2^, or 1 μg/cm^2^, respectively.

Food consumption (S) was determined by the fresh/dry weight method as follows: a fresh corn leaf was cut in half from the main vein and weighted separately; one half was dried at 100 °C for 2 h to calculate fresh/dry weight ratio, calculated as R [34]; the other half, whose fresh weight was S1, was reserved for the feeding experiment. After feeding duration, the residual consumed leaf was dried and weighed as above, and the dry weight was labelled as S2. Food consumption was calculated as: S = S_1_/R − S_2_.

To detect the effects of AMCAL on the physiology of the locust, fourth instar nymphs of similar weight and physiological condition were used to test the effects of AMCAL on locust feeding amount and survival. The increase of locust body weight was recorded individually in treated and control groups. Weight increase rates (WI) were calculated as follows: WI = (W_X_ − W_0_)/W_0_, (W_X_, weight on day x; W0, the day before treatment). The survival rate was calculated by the equation, survival rate (%) = survival numbers/total test numbers.

For the role of antenna or mouthparts in the detection of AMCAL, a dual-choice experiment was performed. Newly molted fourth instar locusts of similar weights were anesthetized on ice for several minutes. Antennae were carefully removed with fine scissors, and then the locusts were fed with fresh corn leaves for 24 h. The locusts were then placed in feeding boxes, each of which contained two pieces of corn leaves of the same size of 4 × 3 cm, one treated with AMCAL in solvent, and the other one treated with solvent alone. The two pieces of corn leaves were placed about 12 cm apart. Feeding response (FR) was defined as follows: a leaf that was ingested, regardless of amount, was scored as 1, whereas no consumption was scored as 0. The experimental duration was about 1 h. When approximately 50% of the control foliage had been consumed, the experiment was terminated, and the feeding amount was determined as described above.

For the dual-choice experiment, the feeding deterrence index (FDI) was calculated using the formula: FDI = (C − T)/(C + T), where C and T indicated control and treated leaf consumption weights, respectively [34,35]. For non-choice experiment, the FDI was calculated using the formula: FDI = (C − T)/C. A regression curve line against feeding deterrence index was created by Graphpad Prism 9 software (Graphpad Software, San Diego, CA, USA). The DC_50_ was defined as the concentration of AMCAL that halved the value of the feeding deterrence index.

The number of individual locusts used in the vivo experiments were 25 locusts for one repetition.

### 2.7. Detecting the Effects of Deficiency of *Lmig*CSPIII by RNAi 

Double-stranded RNA was synthesized using the T7 RiboMAX™Express RNAi System (Promega, Madison, WI, USA), according to the manufacturer’s protocol. Briefly, we designed degenerate primers with a T7 promoter (Table 1) at the 5′ end based on an alignment of multiple LmigCSPIII sequences. The PCR-amplified fragment was purified and used as a template in the transcription reactions. After removing the DNA template and single-stranded RNA using a nuclease, we quantified the newly synthesized dsRNA and adjusted its concentration to 2.5 µg/µL. Locusts were anaesthetized on ice for easier handling during the injection process. dsRNA (2 µL) or DEPC (Diethy Pyrocarbonate) treated water (2 µL, Sangon Biotech) was injected into the dorsal intrasegmental membrane between the third and fourth abdominal segments of newly-molted third or fourth instar locusts using a microsyringe (Narishige, Tokyo, Japan), similarly to previous experiments [36,37,38].

The treatments and calculation methods for detecting the effects of suppression of LmigCSPIII on locusts are similar to in the dual-choice experiment. 

To detect the effects of suppression of *Lmig*CSPIII on the feeding amount or weight increases of locusts, we weighed and recorded the daily amount of remaining leaves after feeding by the mutant (injected with dsRNA of *Lmig*CSPIII) or wild-type locusts (injected with DEPC treated water), and the body weight of each mutant and wild-type locust. 

To detect the effect of suppressing expression of *Lmig*CSPIII on nymphal duration, we created mutant nymphs deficient of *Lmig*CSPIII by injecting with dsRNA of *Lmig*CSPIII into third instar nymphs within 2 days after molting. The wild-types were injected with DEPC treated water only. These two types of locusts were provided with fresh corn leaves every day until the fifth instar; the molting time between the two instars was observed and recorded until the emerged as adults.

To determine the effects of RNAi of *Lmig*CSPIII on its role in detecting AMCAL, we created mutants by injecting with dsRNA of *Lmig*CSPIII into fourth instar nymphs within 2 days after molting. These locusts were then provided with the corn leaves treated with AMCAL or solvent 72 h after injection. The duration of the experiment was 2 h.

### 2.8. Statistical Analyses

Data were analyzed using SPSS 11.5 software (IBM, Chicago, IL, USA). Student’s *t*-test was used to compare control and treatment groups in the dual-choice feeding assay and no-choice *Lmig*CSPIII-deficient growth assay, while one-way analysis of variance (ANOVA), followed by the Dunnett test, was adopted for multiple comparisons of RNA expression levels. A *p*-value of less than 0.05 was considered statistically significant.

## 3. Results

### 3.1. *Lmig*CSPIII Gene Is Expressed in Chemosensory Organs Throughout Development

**Figure 1:**
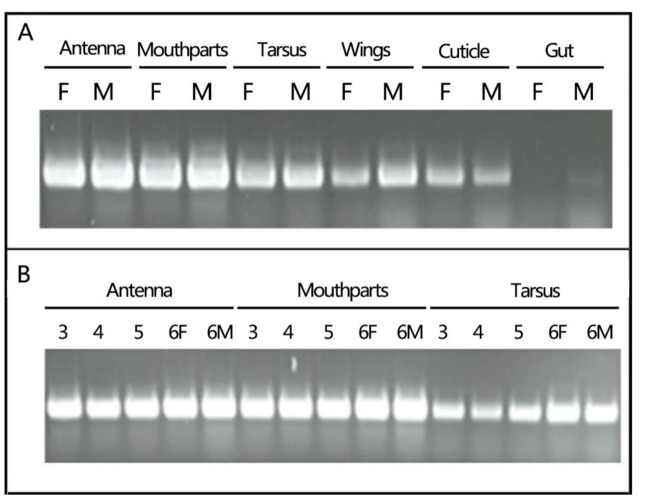
Spatial and temporal expression patterns of *Lmig*CSPIII, as detected by RT-PCR. (**A**) Similar expression profiles of *Lmig*CSPIII shared in several chemosensory organs in new emerged female (F) and male (M) adult *L. migratori*a. (**B**) *Lmig*CSPIII was detected at both nymphal and adult stages in the antenna, mouthparts, and tarsi; 3, 4, 5 represent locust nymphs at third, fourth or fifth instar; six, adults; F, female; M, male.

Using RT-PCR, we examined the expression patterns of *Lmig*CSPIII in selected organs and at different developmental stages. We found that *Lmig*CSPIII was expressed in all the selected organs (except the gut), but at high levels in sensory organs (e.g., antenna, mouthparts, and tarsus) where the chemosensilla are widely distributed (Figure 1A). The gut was the exception, presumably because it contains no chemosensory organs, such as sensilla chaetica. In addition, no sexual dimorphic expression patterns were observed.

*Lmig*CSPIII mRNA exhibited homogeneous expression in antennae, mouthparts, and tarsus, with high transcript levels throughout development (Figure 1B). *Lmig*CSPI, II and III have very similar amino acid sequences compared with CSPs from *Schistocerca gregaria* (*Sgre*CSPI or II, or III, respectively). CSP I and II were expressed similarly in both *L. migratoria* and *S. gregaria* [29]. By contrast, *Lmig*CSPIII gene is detected during all developmental stages (Figure 1B). We hypothesized that *Lmig*CSPIII might function in basic chemoreception in the migratory locust, differently from *Sgre*CSPIII in the desert locust.

**Figure 2:**
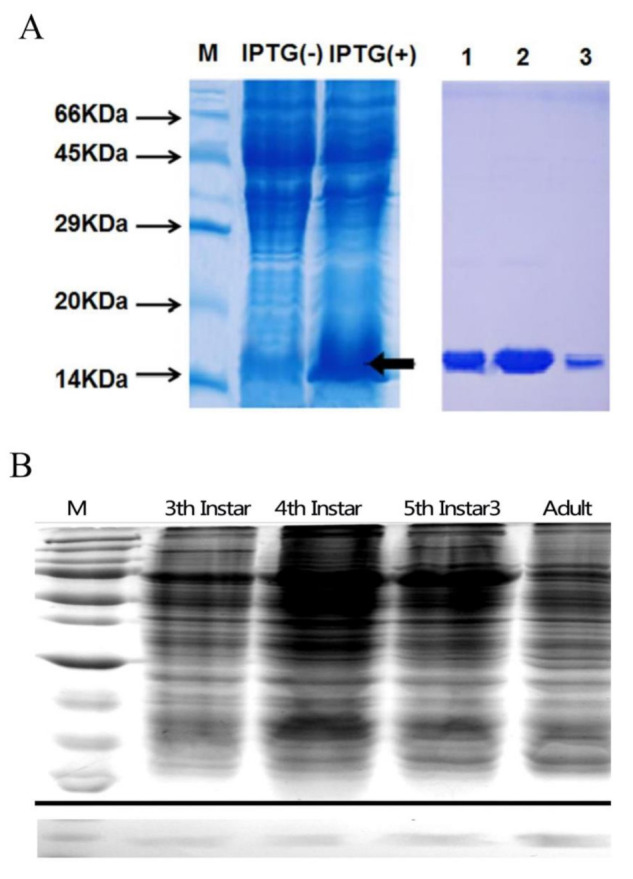
(**A**) Expression and purification of recombinant *Lmig*CSPIII, as analyzed by SDS-PAGE. (Left panel) M, protein molecular weight marker from the top: 66, 45, 29, 20, and 15 KDa. IPTG (−), bacterial cells before IPTG induction. IPTG (+), bacterial cells after IPTG induction. A black arrow indicates the target recombinant protein. (Right panel) Purified recombinant protein with the expected molecular weight of approximately 14 kDa. Proteins were mostly expressed in the supernatant; affinity chromatography was used for purification. The numbers denote the last three fractions of the purification step. (**B**) Western blotting results. Upper, SDS-PAGE of the locust antenna proteins; Lower, corresponding Western blot. Antiserum against LmigCSPIII cross-reacted with locust antenna protein at different developmental stages. M, standard protein marker; 3, 4, 5, 6, as in B.

We expressed *Lmig*CSPIII in BL21 (DE3) *E. coli* cells (Figure 2A). The purified protein was used as antigen to produce antiserum. We then detected the expression patterns of the protein in different developmental stages of the antenna and found that the protein was expressed in the antennae of nymphs and adults (Figure 2B).

### 3.2. *Lmig*CSPIII Has the Highest Binding Affinity to α-Amylcinnamaldehyde

**Figure 3:**
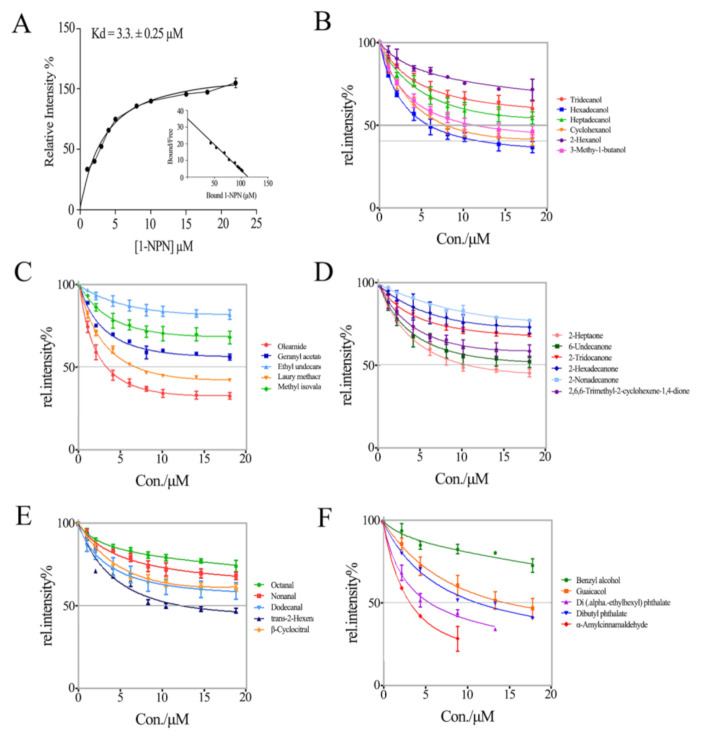
Binding affinity of the recombinant *Lmig*CSPIII to semiochemicals. (**A**) The dissociation curve of *Lmig*CSPIII with the fluorescent reporter, 1-NPN. Relationship between bound 1-NPN (*X*-axis) and bound/free 1-NPN (*Y*-axis) in the system is aligned in a Scatchard plot. (**B**–**F**) Binding affinity of *Lmig*CSPIII to alcohol (**B**), esters (**C**), ketones (**D**), aldehyde (**E**), and aromatic compounds (**F**). Relative intensity indicates fluorescent values at 410 nm. Of all tested chemicals, only those with effectively reduced fluorescent intensity within 20 μm are shown. Each point represents the mean ± S.E.M of three independent replications.

**Figure 4:**
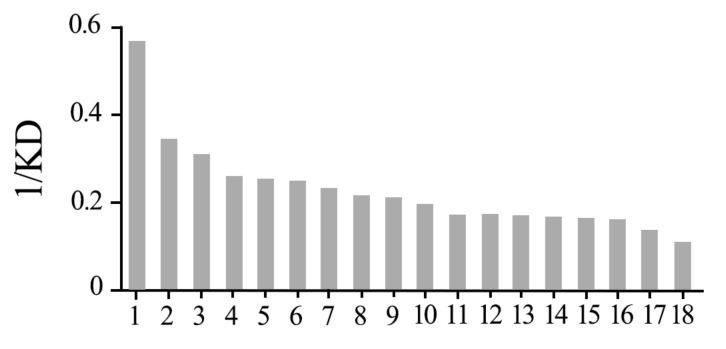
Binding affinities of *Lmig*CSPIII to certain compounds. Only those with an IC_50_ less than 20 µm are regarded as able to bind with *Lmig*CSPIII. Eighteen chemicals were screened and plotted on the chart. KD, dissociation constant. 1-18 represent α-Amylcinnamaldehyde, Oleamide, Diethyl phthalate, Dibutyl phthalate, Benzeneacetonitrile, Guaicacol, Benzaldehyde, Dodecyl 2-methylacrylate, Hexadecanol, Cyclohexanol, 2-Heptaone, DL-sec-phenethyl alcohol, trans-2-Hexenal, 3-Methy-1-butanol, 2,5-Dimetylpyrazine, Phenol, Pentadecanol, Heptadecanol.

To detect the function of *Lmig*CSPIII, we conducted fluorescent competitive binding experiments of recombinant *Lmig*CSPIII with 78 ligands from different sources. The 78 chemicals consists of the aliphatic acids, aliphatic alcohols, aliphatic ketones, aliphatic aldehydes, aliphatic ester derivatives, orbicular compounds, heterocyclic compounds, aromatic compounds, and aliphatic alkane groups. When activated, the strong fluorescence emission (at 295 nm) of the recombinant *Lmig*CSPIII was effectively quenched by N-phenyl-1-naphthylamine (1-NPN), indicating that the protein has a hydrophobic core [32]. The binding affinity of *Lmig*CSPIII to other compounds was then monitored with a competitive binding assay using 1-NPN as a reporter. The dissociation constant of *Lmig*CSPIII/1-NPN was 3.30 µM (Figure 3A). Among the 78 tested compounds, aromatic bulky compounds such as AMCAL and 1-aminoanthracene, showed the strongest affinity toward the recombinant *Lmig*CSPIII. In contrast, most linear aliphatic alkanes and their derivatives could only replace1-NPN from the binding pocket at very high concentrations (Figure 3B–E). Nevertheless, the larger compounds (C15–C17) had stronger binding affinities, with dissociation constants ranging from 4.77 ± 0.20 µM to 9.15 ± 0.46 µM. In addition, 3-Methyl-1-butanol, which has only four carbon atoms and is present in locust body volatiles, also exhibited a higher binding capacity (6.02 ± 0.17 µM). Two other body volatile components, trans-2-hexenal and 2-heptanone, were able to displace 1-NPN from *Lmig*CSPIII at a concentration of 10 µM, indicating a limited binding affinity. Finally, oleamide, which can be extracted from locust wings, also exhibited a quite high binding affinity. Other compounds, such as those from host plants, the locust body or fecal volatiles, could not effectively replace 1-NPN from the protein. Most importantly, among the compounds with higher affinity to the protein, AMCAL had the highest affinity (Figure 3F and Figure 4). Several reports have indicated that AMCAL occurs naturally in plants that are not hosts of *L. migratoria* [39]. Thus, we inferred that *Lmig*CSPIII might mediate recognition of this botanical secondary metabolite.

### 3.3. A-Amylcinnamaldehyde Is a Locust Antifeedant

As the chemical AMCAL has highest affinity to *Lmig*CSPIII, we then asked how this compound affects locusts. We carried out bioassays to determine whether this compound could be detected by chemosensory organs, such the antenna or mouthparts, and its bioactivity to locusts.

**Figure 5:**
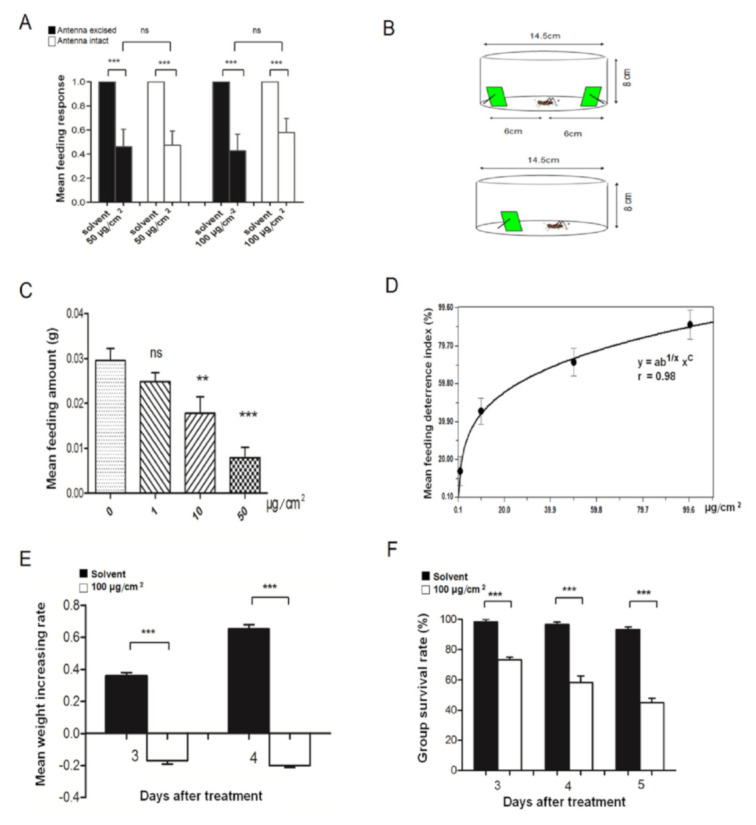
Gustatory perception and biological effects of AMCAL. (**A**) Feeding response of fourth instar nymphs with or without antennae to control and treated leaves (***, *p* < 0.001, *t*-test, *n* ≥ 20; dual-choice test). (**B**) Devices for feeding experiments. Upper, the box used in the dual-choice test; lower, the box used for the non-choice test. (**C**) The mean amount of fourth instar nymphs feeding on leaves spread with AMCAL (**, *p* < 0.01; ***, *p* < 0.001, ANOVA, Dunnett test; *n* ≥ 20; non-choice test). (**D**) Nonlinear regression analyses against feeding deterrence index of fourth instar nymphs (*n* ≥ 20; dual-choice test). The mean increase rate of body weight (**E**), or survival rate (**F**) of third instar nymphs after consumption of leaves containing AMCAL at 100 μg.cm^−2^ (***, *p* < 0.001, *t*-test; *n* = 15–20; non-choice test). Each point represents the mean ± S.E.M of three independent replications. ns; no statistically significant difference.

The feeding response (FR) patterns of locusts with intact or excised antennae were similar in a dual-choice feeding assay. Nymphs from both groups tended to consume the control leaves (FR = 100%) rather than treated leaves (50 μg/cm^2^, FR = 45% for antennae excised; FR = 47% for antennae intact). This result indicated that removal of the antennae, which are major olfactory organs, had little impact on feeding-choice patterns. Considering the nearly equal possibility of first-bite, the higher FR to control leaves is more likely to derive from gustatory aversion after initial consumption, rather than a second feeding-choice; however, it was not a result of olfactory repulsion. If the olfactory decision was decisive, then the FR to treated leaves would have declined sharply. When the concentration of AMCAL on treated leaves was increased to 100 μg/cm^2^, similar FR profiles were obtained (Figure 5A), suggesting that the amount of chemical applied to the leaves was sufficient to elicit a gustatory decision. 

We next assessed the effect of AMCAL on the amount consumed. The mean amount consumed and feeding deterrence index were measured to obtain the antifeedant potency against fourth instar nymphs (Figure 5C,D). The mean consumption of food containing the chemical decreased in a concentration-dependent manner. Significant feeding inhibition began to occur at a concentration of 10 μg/cm^2^ (*p* < 0.01, Dunnett test), although a slight and insignificant decrease was observed beginning at a concentration of 1 μg/cm^2^ (*p* > 0.05; Dunnett test). A high-degree fitting curve was obtained between the feeding deterrence index and concentration of the compound on leaves, using regression analyses (Figure 5D); from this curve, the DC50 was predicted to be 12 μg/cm^2^. 

An antifeedant may generate physiological disruption in insects. Therefore, we conducted an experiment to check whether this compound affects the physiological characters of locusts. We found that the weight increase of nymphs at third instar feeding on the leaves containing AMCAL at the rate of 100 μg/cm^2^ was significantly less than that of the control group after 3 or 4 days of treatment (Figure 5E, *p* < 0.001, *t*-test). In addition, the survival rate of the nymphs feeding on leaves containing AMCAL at the same rate decreased significantly from days 3–5 after treatment, compared with control groups (Figure 5F, *p* < 0.001, *t*-test). Taken together, our results indicated that AMCAL is an effective antifeedant that functions predominantly through the gustatory system.

### 3.4. *Lmig*CSPIII Deficient Mutants Have Decreased Sensitivity to α-Amylcinnamaldehyde

**Figure 6:**
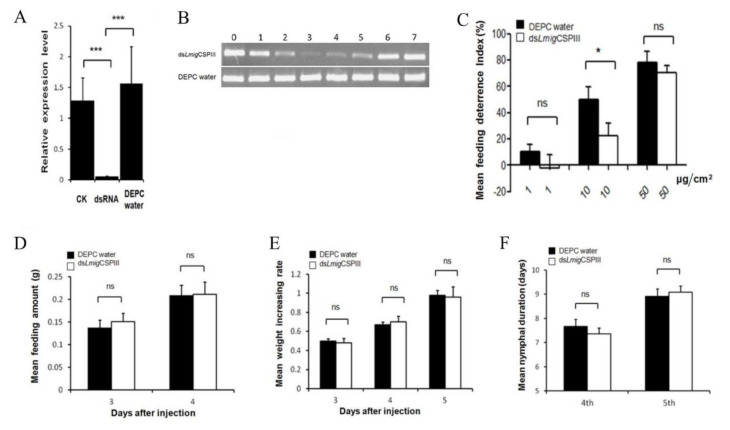
Suppression of *Lmig*CSPIII expression decreased the locust’s perception of AMCAL. (**A**) Silencing efficiency was determined by qRT-PCR of mouthparts tissues on day 3 of the fourth instar nymphs (***, *p* < 0.001, AVONA, Dunnett test; *n* = 5–10). (**B**) Efficiency of RNA interference, as assessed by RT-PCR after injection with ds*Lmig*CSPIII. 0, represents non-injection; 1, 2, 3, 4, 5, 6 and 7 represent day(s) after injection with dsLmigCSPIII or DEPC water. (C) Locusts with *Lmig*CSPIII-knockdown were less sensitive to the antifeedant than the H_2_O-injected controls (*, *p* < 0.05, *t*-test; *n* ≥ 20; dual-choice test). (**D**–**F**) Feeding amount, weight increase and nymphal duration were not influenced by *Lmig*CSPIII suppression (*t*-test; *n* = 15–20; non-choice test). Each point represents the mean value ± S.E.M (standard error of mean) of three independent replications. ns; no significant difference.

We next investigated whether *Lmig*CSPIII is involved in detecting AMCAL in locusts. We performed microinjection of dsRNA to interfere with the expression of the protein, and then checked the feeding deterrence index (FDI) using a dual-choice feeding assay. First, PCR experiments showed that the expression of *Lmig*CSPIII gene was almost completely suppressed in the mouthparts of injected locusts on the third day after dsRNA injection (*p* < 0.001, Dunnett test), but was normally expressed in H_2_O-injected and non-injected controls (Figure 6A,B). The *Lmig*CSPIII deficient locusts were viable and fertile, with no gross morphological or locomotion deficits compared with control locusts (data not shown). 

We also found that locusts injected with dsRNA of *Lmig*CSPIII were less responsive to AMCAL-painted leaves at a concentration of 10 μg/cm^2^ compared with the control group (FDI = 22% vs. 50%, *p* < 0.05, *t*-test, Figure 6C). However, the FDI of the RNAi group did not significantly decrease at an AMCAL concentration of 1 μg/cm^2^ (−2%) or 50 μg/cm^2^ (69%) (*p* > 0.05, *t*-test). These results suggested that *Lmig*CSPIII could mediate the sensitivity of locusts to AMCAL over a certain range of concentrations. 

Finally, we examined whether *Lmig*CSPIII deficiency has other effects on locusts’ physiological characters. Our results showed that suppression of *Lmig*CSPIII did not change either the feeding rate or weight gain of the locusts (Figure 6D,E, *p* > 0.05, *t*-test). The duration of the fourth and fifth instars did not change significantly either (Figure 6F, *p* > 0.05, *t*-test). These data suggest that *Lmig*CSPIII does not play a role in determining the feeding amount of host-plant and in physiological development. Thus, *Lmig*CSPIII is responsible solely for the sensitive detection of antifeedants.

## 4. Discussion

Host–plant discrimination requires the detection of plant secondary metabolites and is biologically and ecologically significant to herbivores. To avoid noxious chemicals, they will reduce or terminate food ingestion when they distinguish unpalatable diets, which are important for their survival and development [40]. Aversion to such feeding deterrents, or antifeedants, indicate that specific recognition mechanisms, particularly the gustatory system, are involved, although it varies among herbivorous species. 

Chapman pointed out that the interaction of phagostimulants and deterrent chemicals affect the selection of food by insects [41]. The effects of such interactions in the selection of hosts were supported by an extensive series of experiments with *L. migratoria* and *S. gregaria* [5,42]. Most studies focused on molecular mechanisms of phagostimulants and bitters of animals and insects, with few focusing on antifeedants. 

Gene expression profiles and protein binding properties of chemosensory proteins in insects have revealed the important function of this protein [21,43,44]. Our results indicated that *Lmig*CSPIII is expressed in organs that are covered with chemosensilla and tactile sensilla and may function in perceiving chemical stimuli. However, our findings, in combination with previous immunocytochemistry localization and single unit electrophysiological studies, do not support the hypothesis that CSPs participate in olfaction-related behaviors [27,45,46]. Ligand-binding experiments demonstrated that CSPs have relatively narrow binding specificities [32]; their crystal structures have revealed binding pocket-bearing domains or binding sites, as well as conformational plasticity, indicating that CSPs most likely function by interacting with chemicals [47,48]. Our binding experiments showed that *Lmig*CSPIII has an extraordinarily high binding affinity to the plant secondary metabolite, AMCAL. In addition, the lower expression levels of *Lmig*CSPIII after dsRNA injection persisted for approximately five days, even after the molt. During this period, the amount of food ingested or developmental duration of *Lmig*CSPIII knockdown nymphs did not show any differences from the wild-type control. These results indicated that *Lmig*CSPIII is not involved in locust development and growth. Several previous studies suggested that CSPs might have non-chemosensory functions [22,23,24]. However, we found that the *Lmig*CSPIII-deficient mutants exhibited reduced sensitivity to AMCAL at a range of concentrations, providing the first evidence of the participation of a CSP in contact chemosensation. 

The AMCAL, which was identified as a binding ligand of *Lmig*CSPIII, occurs naturally in plants, such as jasmine and cinnamon, which are not host plants for *L. migratoria* [39]. The feeding response assay revealed that gustatory detection to this compound is the main pathway, rather than olfactory perception. This agreed with Chapman’s suggestion that locusts recognize non-hosts via the palps of mouthparts [41], and Sinoir’s result that locust biting was induced by the stimulation of mechanoreceptors on the labrum and galea [49].

The effectiveness of feeding deterrence of AMCAL was determined by DC_50_ (12 μg/cm^2^) using nonlinear regression analysis. This DC_50_ is higher than that of a common antifeedant against locusts, azadirachtin, whose value is 1 μg/cm^2^. However, the effects of AMCAL on body weight increase and mortality of locusts in our study indicated its toxic properties. Therefore, AMCAL can be regard as an antifeedant for *L. migratoria*. Similar effects of antifeedants have been found in noctuid caterpillars and other insects [34,35]. In the desert locust, the most potent antifeedant, azadirachtin, reduces fitness and fertility, and interferes with molting at a concentration of 7 µg/g body weight [50]. Even host plants contain some deterrents. Woodhead [51] reported that alkanes with chain lengths of 19, 21, 23 and 24 carbon atoms extracted from seeding sorghum are deterrents for locusts1. Bernays [42] and Chapman [41] suggested that the responses of grasshoppers to plant secondary compounds fall into five classes2. According to their standards, AMCAL belongs to Class I, for which food intake is reduced as the concentration increases.

We only observed significant effect at 10 µg/cm^2^, and we speculate that locust can detect AMCAL at certain concentration through *Lmig*CSPIII, and 10 µg/cm^2^ and 50 µg/cm^2^ is either too low or too high concentration for locust. When at high concentration, the AMCAL can reach receptor directly without the delivery of *Lmig*CSPIII.

Herbivores’ dietary preferences have evolved from long-term interactions with plants with idiosyncratic sensing systems to identify antifeedants. Even closely related phytophagous animals may respond differently to the same antifeedant [52]. *Lmig*CSPIII in *L. migratoria* and CSPSgre-III-1 in *S. gregaria* are conserved orthologs between the two species. We deduced that the function of *Lmig*CSPIII is similar to that in CSPSgre-III-1. However, our western blotting experiment revealed the presence of mature *Lmig*CSPIII throughout the developmental stages of the migratory locust, *L. migratoria*, which is different from CSPSgre-III-1, which was only detected in adulthood in the desert locust, *S. gregaria*. This implied that CSPIII has species-specific characteristics, although the protein may have the same general role. The two closely-related species, *L. migratoria* and *S. gregaria*, have distinct dietary habits: *L. migratoria* is an oligophagous herbivore, with a narrow host–plant spectrum, and eats grass and sedge plants; while *S. gregaria* is polyphagous, and has a very broad host–plant spectrum. *Lmig*CSPIII is demonstrated to be involved in detecting antifeedant, as a limiting factor for feeding in *L. migratoria*; therefore, we hypothesized that *Sgre*CSPIII may conduct a similar role, but the lack of expression in nymphal stage may mean that the desert locust has less limitation in feeding.

OBPs and CSPs have quite similar amino acid sequences and three-dimensional structures; we hypothesized that they might share a homologous mode in chemoreception because of convergent evolution. CSPs in locusts can be detected in chaetic sensilla lymph and their function is proposed to transport taste molecules and thus assist signal cascade transduction in the peripheral nervous system [4,53]. Therefore identification of other elements in the gustatory neural circuitry, such as gustatory receptors may reveal the whole molecular mechanisms of peripheral nervous events in detecting antifeedant. Because functional analysis toward insect gustatory receptors have shed light on how gustatory chemical stimuli are transduced into potential actions of sensory neurons and their indispensability in feeding behaviors [54,55,56,57]. Therefore, the gustatory receptors involved in detecting antifeedants require further research.

## 5. Conclusions

In the study of the function of chemosensory protein (CSP), we demonstrated that *Lmig*CSPIII in *Locusta migratoria*, which is widely distributed and highly expressed in sensory organs, was involved in detecting an antifeedant. *Lmig*CSPIII exhibits highest binding affinity to α-amylcinnamaldehyde (AMCAL), a natural compound from non-host plant which was subsequently demonstrated to be an effective antifeedant to locusts in feeding bioassay. RNA interference of *Lmig*CSPIII showed reduced sensitivity at certain concentration of AMCAL, but did not change in their physiological development or food consumption. In summary, our results indicate that this chemosensory protein was involved in antifeedant detection.

## Figures and Tables

**Table 1:** Specific primers used in this study.

	Primer Names	Sequences (5’-3’)
RT-PCR	*Lmig*CSPIII-s *Lmig*CSPIII-as	ACGCCTCCTCAAGTCCTACA AGCTGTTTCACTTATTCACAGAGT
qPCR	*Lmig*CSPIII-s *Lmig*CSPIII-as	AAGGGGTGGGAGACGGCCTG CAGCTCCTCCCCAACGACAGC
ds*Lmig*CSPIII	*Lmig*CSPIII-s	TAATACGACTCACTATAGG AAGGGGTGGGAGACGGCCTG
	*Lmig*CSPIII-as	TAATACGACTCACTATAGG CAGCTCCTCCCCAACGACAGC
Clone	*Lmig*CSPIII-s	CATATAGGGCCACTCAGGACCCGCTG *Nde1*
	*Lmig*CSPIII-as	GAATTCTCAGAAGTTGATGCCGCGGTG *EcoR1*
Control	*Lmig*Actin-s *Lmig*Actin-as	GCAAAGCTGGCTTCGCCG ATGTTCCTCGGGCGCCAC

T7 polymerase promoter sequence are underlined for ds*Lmig*CSPIII synthesis. Enzyme restriction sites are underlined.

**Table 2:** Compounds used as ligands.

Ligands	
**Aliphatic acid**	**Aliphatic ester derivatives**
Butanoic acid	Methyl isovalate
Pentadecanoic acid	Ethyl cis-3-hexenoate
Palmitic acid	Ethyl caprylate
**Aliphatic alcohols**	Ethyl nonanoate
3-Methy-1-butanol	Ethyl caprate
2-Hexanol	Ethyl undecanoate
3-Hexanol	Ethyl laurate
3- Octanol	Ethyl tridecanoate
5-Nonanol	Ethyl myristate
Linalool	Dodecyl 2-methylacrylate
3,7-Dimethyl octanol	Ethyl palmitate
*cis*-3,7-Dimethyl-2,6-octadien-1-ol	Oleamide
10-Undecylenylalcohol	Ethyl sterate
Undecanol	Geranyl acetate
2,2,4-Trimethyl-3-nonanol	**Orbicular compounds**
Tridecanol	Cyclohexanol
Dodecanol	2,5-Dimethyl cyclohexanone
Tetradecanol	α-Pinene
Pentadecanol	2,6-Dimethyl cyclohexanone
Hexadecanol	2,6,6-Trimethyl-2-cyclohexene-1,4-dione
Heptadecanol	**Heterocyclic compounds**
1-Hydroxyoctadecane	2,5-Dimetylpyrazine
Guaicacol	β-Cyclocitral
**Aliphatic ketones**	1-Aminoanthracen
2-Heptaone	**Aromatic compounds**
6-Dimethyl-4-heptanone	Phenol
2-Undecanone	Benzaldehyde
6-Undecanone	Benzyl alcohol
2-Dodecanone	DL-sec-phenethyl alcohol
2-Tridocanone	Benzeneacetonitrile
2-Pentadecanone	4-Tert-butylphenol
2-Hexadecanone	Dimethyl phthalate
2-Heptadecanone	Diethyl phthalate
2-Octadecanone	α-Amylcinnamaldehyde
2-Nonadecanone	Benzyl benzoate
6-Methyl-5-Hepten-2-one	Dibutyl phthalate
**Aliphatic aldehydes**	Di (.alpha.-ethylhexyl) phthalate
trans-2-Hexenal	**Aliphatic alkane**
Hexanal	1-Undecene
Octanal	1-Hexadecene
Nonanal	1-Nonadecene
Decanal	
Undecylic aldehyde	
Dodecanal	
Tridecanal	
